# Three Holy Men Get Haircuts: The Semiotic Analysis of a Joke

**DOI:** 10.5964/ejop.v12i3.1042

**Published:** 2016-08-19

**Authors:** Arthur Asa Berger

**Affiliations:** a Broadcast and Electronic Communication Arts at San Francisco State University, San Francisco, CA, USA; Department of Psychology, University of Western Ontario, London, Canada

**Keywords:** humor, techniques, jokes, syntagmatic, paradigmatic, Jewish, masochism

## Abstract

This article deals with a typology of 45 techniques of humor that I found when doing research on the mechanisms that generate humor in texts, lists the techniques and applies them to a Jewish joke. It references the work of Vladimir Propp on folktales as analogous in that both are concerned with mechanisms in text that generate meaning. It also deals with four theories about why people find texts humorous, defines the joke as a short narrative with a punch line that is meant to generate mirthful laughter and defines Jewish humor as being about Jewish people and culture as told by Jewish people. It offers a paradigmatic analysis of the joke, and offers some insights into why Jewish people developed their distinctive kind of humor. This article is an enhanced and expanded version of an article which was published in a Chinese semiotics journal (doi:10.1515/css-2015-0022).

This article^1^ is about different approaches to humor and the difference between so-called “why” theories of humor and a list of 45 mechanisms that I suggest generate humorous responses to texts. Let me begin with an overview of the four dominant theories of humor. These theories claim to explain why people laugh.

## The Four Why Theories of Humor

The first theory argues that humor is based on a sense of *superiority.* Aristotle said humor is based on (McKeon, 1941, p. 1459) “an imitation of men worse than average; worse, however, not as regards any sort of fault, but only as regards one particular kind, the Ridiculous, which is a species of the Ugly. The Ridiculous may be defined as a mistake or deformity not productive of pain or harm to others.” Thomas Hobbes explained that “the passion of laughter is nothing else but the sudden glory arising from a sudden conception of some eminency in ourselves by comparison with the infirmity of others, or with our own formerly.” (Quoted in Arthur Koestler, 1949, *Insight and Outlook,* p. 56).

The second “why” theory, and most widely held one, is *incongruity* theory which argues that laughter is created when there is a difference between what we expect and what we get. The punch line in jokes generates an incongruity that we find amusing. Schopenhauer describes what we call the incongruity theory as follows (Piddington, 1963, pp. 171-172) “The cause of laughter in every case is simply the sudden perception of the incongruity between a concept and the real object which have been thought through it in some relation, and laughter itself is just the expression of this incongruity.” In jokes, the sudden perception that Schopenhauer mentions is caused by the punch lines which generate this recognition of an incongruity. In a good joke, we don’t know what to expect in the way of a punch line.

The third “why” theory is the *psychoanalytic* theory of humor which suggests that humor is primarily a form of masked aggression.” As Freud wrote in his book, *Jokes and Their Relation to the Unconscious* (Freud, 1963, p. 101) “and here at last we can understand what it is that jokes achieve in the service of their purpose. They make possible the satisfaction of an instinct (whether lustful or hostile) in the face of an obstacle that stands in its way.” (Freud, 1963, p. 101). “The wonderful thing about humor, from a psychoanalytic perspective, is that when we hear a joke we can participate in the aggression without any sense of guilt.

The fourth “why” theory ties humor to *communication paradoxes* and suggests that humor results from the use of paradox, play and the resolution of logical problems. As William Fry wrote in his book *Sweet Madness* (Fry, 1963, p. 158) “During the unfolding of humor, one is suddenly confronted by an explicit-implicit reversal when the punch line is delivered…Inescapably, the punch line combines communication with meta-communication.” In the final analysis, these theorists argue that what goes on in jokes may be too complicated for us to understand at our present level of development.

The problem with these theories is that they don’t explain how humor arises in the events that take place in jokes. For example, incongruity theorists deal with surprises in jokes. Since all jokes contain punch lines, which generate unexpected resolutions to jokes, all my 45 techniques can be subsumed under the incongruity theory of humor. But there is a difference between talking about incongruity and about the various techniques I deal with in my typology: insult, facetiousness, exaggeration and so on. Now that I’ve discussed the four “why we laugh” theories, let me say something about how I developed my list of the 45 techniques of humor.

In Vladimir Propp’s *Morphology of the Folktale* he offers us thirty one functions that describe actions by characters who play an important role in folktales. These functions help us understand how narrative texts work. Some typical functions are interdiction, violation, trickery, and the receipt of a magical agent. Propp chose to focus on functions of characters in folktales because other approaches, such as studying themes or kinds of heroes and heroines, didn’t work. He defined a function as (Propp, 1968, p. 21) “An act of a character, defined from its point of view of its significance for the course of action.” These functions, he added, are stable and their number is limited. I had not read Propp’s *Morphology of the Folktale* when I started doing my research into what I describe as the techniques of humor. But after I read Propp’s book (I had already finished my research and found 45 techniques that are similar in nature to his functions) I considered using it as a title for one of my books on humor, and calling it *The Morphology of the Joketale.* I settled for *An Anatomy of Humor.*

I have been interested in humor for many years and have dealt with it in a scholarly way from my 1965 dissertation on a humorous comic strip, *Li’l Abner,* to my books such as *An Anatomy of Humor, The Genius of the Jewish Joke, Blind Men and Elephants,* and *The Art of Comedy Writing,* all of which appeared in the 1970s. In the early seventies, I did some research on what it is that generates humor in texts and found 45 techniques that, I argue, inform all humor. My first article in which I dealt with these techniques was “Anatomy of a Joke,” published in the *Journal of Communication* in the summer of 1976. I argue that these techniques are at work in all humorous texts to generate mirthful laughter. They tell is what makes us laugh which is different from why we laugh. Now let me offer a great Jewish joke.

## A Priest, An Imam and a Rabbi Get a Haircut

This joke was told to me by a Jewish friend from Israel. I found it very funny and had a good laugh when I heard it but some people who are not Jewish might not “get” it.

A barber is sitting in his shop when a priest enters. “Can I have a haircut?” the priest asks. “Of course,” says the barber. The barber than gives the priest a haircut. When the barber has finished, the priest asks “How much do I owe you?” “Nothing,” replies the barber. “For you are a holy man.” The priest leaves. The next morning, when the barber opens his shop, he finds a bag with one hundred gold coins in it. A short while later, an Imam enters the shop. “Can I have a haircut?” he asks. “Of course,” says the barber, who gives the Imam a haircut. When the barber has finished, the Imam asks “How much do I owe you?” “Nothing,” replies the barber. “For you are a holy man.” The Imam leaves. The next morning, when the barber opens his shop, he finds a bag with a hundred gold coins in it. A bit later, a rabbi walks in the door. “Can I have a haircut?” the rabbi asks. “Of course,” says the barber, who gives the rabbi a haircut. When the haircut is finished, the rabbi asks, “How much do I owe you?” “Nothing,” replies the barber, “for you are a holy man.” The rabbi leaves. The next morning, when the barber opens his shop, he finds a hundred rabbis.

The problem with the “why” theories is they don’t deal with the mechanisms in the joke I’ve just “told”—and we will be dealing with jokes here—short texts that generate the humor. I should point out that supporters of the various “why” theories spend a lot of time arguing with supporters of the other “why” theories about which theory is best. But what is important, from my perspective, is that the “why” theories don’t deal with the specifics of jokes to explain what it is, in a given joke, that evokes mirthful laughter.

Superiority theorists would say we feel superior to the rabbi and the hundred rabbis who are crowded in the barbershop, hoping to get a free haircut. Incongruity theorists would say we are surprised by the punch line, though anyone familiar with Jewish humor might possibly have been able to anticipate the kind of resolution we find in the joke. Psychoanalytic critics would say the joke allows guilt-free aggression against Jews, who are the main protagonists in the joke and the subject of the punch line, and communication theorists would say the resolution is ultimately paradoxical and involves a communication, the punch line, and a meta-communication— laugh, but don’t take this story seriously because it is a joke. But these “why” theories don’t adequately explain what is going on in the joke.

Rather than arguing which “why” theory is best, I chose a different path. As the result of a large research project I conducted—a content analysis using all the books I had in my house with humorous content: joke books, books of folklore, comic strips, cartoons, humorous poems, theatrical comedies, humorous short stories, etc., etc. with a focus on what it was, in each text I examined, that was funny and that generated laughter. I came up with a list of 45 techniques which, I suggest, in various combinations, can be found in jokes and all other forms of humor. These techniques, we might say, are the DNA of humor. We often find two or three, or more, of these techniques in a joke. These techniques, I claim, inform humor from different time periods (for example, Plautus used them in his Roman comedies, Shakespeare used them in his comedies and Ionesco used them in his play *The Bald Soprano*) and in different cultures. I define and offer examples of humorous texts with each of the 45 techniques and then apply the techniques to humorous texts in my books *An Anatomy of Humor* and *The Art of Comedy Writing.*

## What Makes Us Laugh: The 45 Techniques Found in Humor

After I came up with my list, I discovered that the techniques could be classified into four categories: jokes involving *language*, jokes involving *logic*, jokes involving *identity* and jokes involving gestures and similar *actions*.

**Table:** 45 Techniques of Humor by Category

Language Allusion Bombast Definition Exaggeration Facetiousness Insults Infantilism Irony Misunderstanding Over literalness Puns/Wordplay Repartee Ridicule Sarcasm Satire	Logic Absurdity Accident Analogy Catalogue Coincidence Comparison Disappointment Ignorance Mistakes Repetition Reversal Rigidity Theme/Variation Unmasking	Identity Before/After Burlesque Caricature Eccentricity Embarrassment Exposure Grotesque Imitation Impersonation Mimicry Parody Scale Stereotype	Action Chase Slapstick Speed

In order to make these techniques easier to apply, I put them into alphabetical order and numbered them.

**Table:** Techniques of Humor in Alphabetical Order

1. Absurdity 2. Accident 3. Allusion 4. Analogy 5. Before and After 6. Bombast 7. Burlesque 8. Caricature 9. Catalogue 10. Chase Scene 11. Coincidence 12. Comparison 13. Definition 14. Disappointment 15. Eccentricity 16. Embarrassment 17. Exaggeration 18. Exposure 19. Facetiousness 20. Grotesque 21. Ignorance 22. Imitation 23. Impersonation 24. Infantilism 25. Insults 26. Irony 27. Literalness 28. Mimicry 29. Mistakes 30. Misunderstanding 31. Parody 32. Puns 33. Repartee 34. Repetition 35. Reversal 36. Ridicule 37. Rigidity 38. Sarcasm 39. Satire 40. Scale, Size 41. Slapstick 42. Speed 43. Stereotypes 44. Theme and Variation 45. Unmasking

I will show how these techniques function in the joke about a priest, an imam and a rabbi who go to a barbershop to get a haircut. But first, we must decide what a joke is and then what makes a joke a Jewish joke.

## Defining the Joke

I will define a joke as “a short narrative, with a punch line, meant to evoke mirthful laughter.”

The narrative may have a number of events in it but if it is a joke, it will always have a punch line that is meant to generate mirthful laughter. The structure of the typical joke is shown below:

**Table:** 

A →	B →	C →	D →	E →	F (punch line)
					↓
					G Mirthful laughter

This diagram shows that jokes are short narratives and it offers an x-ray of the syntagmatic nature of this kind of text. With this definition of the joke in mind, let me use my list of techniques to analyze the priest, imam and rabbi joke.

## Techniques of Humor in the Priest, Imam and Rabbi Joke

What follows are my suggestions about which techniques of humor are found in this joke. It is not unusual for a joke to make use of a number of different techniques.

### Technique 44: Theme and Variation (Logic Humor)

The first technique we find in the joke is 44, Theme and Variation. In *The Art of Comedy Writing* I define theme and variation as follows (Berger, 1997, p. 44):

By theme and variation I refer to the technique comedy writers use to take some matter (a belief, an activity) and show how different nationalities, religions, occupations, members of social classes, etc. vary with regard to this belief or activity. Part of the humor here comes from seeing how the theme is varied by the different groups, and by the way this technique plays with stereotypes people have of the different groups.

There are three holy men and for most of the joke we find them doing the same thing—getting a haircut, asking to pay for the haircut but being told by the barber the haircut was free, and, for two of them, leaving the barber a hundred gold coins the next day. The three holy men are from different religions and the third holy man, the rabbi, doesn’t leave a hundred gold coins but a hundred rabbis. That is the variation.

### Technique 19: Facetiousness (Linguistic Humor)

In *An Anatomy of Humor* I define facetiousness as follows (Berger, 1993, p. 34):

Facetiousness is generally taken to mean a joking, nonseries us of language. There is an element of ambiguity, for the person does not really mean (or take seriously) what he or she says and this must be communicated one way or another…Facetiousness is similar to irony, but is weaker. In both techniques we must “read” or “decode” the message; in irony there is a reversal, in facetiousness there is a discounting.

I understand facetious to mean a jesting, frivolous, nonserious use of language. The idea of having a hundred rabbis packed into a small barbershop is far-fetched and the joke’s humor is based, in large measure, on the ridiculous nature of the idea.

### Technique 1: Absurdity (Logic Humor)

I deal with absurdity in my *An Anatomy of Humor* and explain (Berger, 1993, p. 19):

Absurdity and its related forms—confusion and nonsense—seems to be relatively simple, but it is not, and its effects may be quite complicated, as Freud pointed out in his discussion of nonsense humor. Absurdity works by making light of the “demands” of logic and rationality. This absurdity doesn’t necessarily take the form of silliness (though in many children’s jokes it does) but may be an example of a relatively sophisticated philosophical position….We all seem to need to impose our sense of logic and order on the world, and when we come across situations or instances where our logic doesn’t work, we react by being puzzled and, in certain cases, amused.

I usually reserve the technique of absurdity to deal with the kind of plays one finds in the theater of the absurd, but it is reasonable to suggest that the idea of packing a hundred rabbis into a barbershop is absurd and that this absurdity helps generate the humor. Absurdity, which is based on logic and irrationality is not the same as facetiousness, which is based on a certain attitude.

### Technique 43: Stereotypes (Identity Humor)

My discussion of stereotypes, found in *An Anatomy of Humor,* relies on sociological theories about the subject. As I explain in my discussion of this technique (Berger, 1993, pp. 52-53):

Jokes involving stereotypes can be described as generalized insults—attacks on races, religions, ethnic groups, etc. but there is more to the humor of stereotypes than that. Stereotypes are useful to writers and comedians because they are instant (pseudo) “explanations” of behavior and they enable people to understand “motivation”….Stereotypes are, from a sociological point of view, group-held notions people have about other groups. Stereotypes can be negative, positive or mixed, but in all cases they are extreme over-simplifications and generalizations.

The joke also alludes to the stereotype that Jews are cheap—a stereotype that is widely held but also quite inaccurate. Instead of a hundred gold coins, the barber finds a hundred rabbis.

### Technique 14: Disappointment (Logic Humor)

In my description of the humor of disappointment and defeated expectations in *An Anatomy of Humor* I write (Berger, 1993, p. 31):

The technique of disappointment involves leading people on about something and then denying them the logical consequences they expect. It is very similar to teasing and is funny to the extent that we find minor disappointments amusing. A good deal depends upon the frame or situation in which the disappointment is staged.

The structure of the barbershop joke, with the first two holy men leaving a hundred gold coins, sets the listener of the joke up to expect that the rabbi will also leave a hundred gold coins. Instead, he “leaves” a hundred rabbis. This humor is based upon defeated expectations.

We can say the “formula” for this joke (that is, the techniques used in it) is: 44-19-43-14. Reducing a joke to a formula is, in itself, humorous. There are, in fact, jokes that use the idea of jokes having numbers to differentiate them from other jokes. For example, consider the following joke:

At a conference of comedians, all the comedians know all the jokes so they now tell jokes by referring them to number. A comedian stands up and says “35-16-9-45” but nobody laughs. A comedian in the audience turns to a friend and says “he never could tell a joke well.”

Now let us turn to a paradigmatic analysis of the priest, imam and rabbi joke.

## The Paradigmatic Structure of the Priest, Imam and Rabbi Joke

In the “Introduction to the Second Edition” of Propp’s *Morphology of the Folktale* (Propp, 1968), Alan Dundes writes (1968, p. xi):

There seems to be two distinct types of structural analyses in folklore. One is the type of which Propp’s *Morphology of the Folktale* is the exemplar par excellence. In this type, the structure or formal organization of a folkloristic text is described following the chronological order of the linear sequences of elements in the text….Following Lévi-Strauss (1963, p. 312) this linear sequential structural analysis we might term “syntagmatic” structural analysis….The other type of structural analysis in a folklore seeks to describe the pattern (usually based upon an a priori binary principle of opposition) which allegedly underlies the folkloristic text. This pattern is not the as the sequential structure at all. Rather, the elements are taken out of the “given” order and are regrouped in one or more analytic schema.

It was Claude Lévi-Strauss who suggested that the paradigmatic analysis of a text showed what it means in contrast to the syntagmatic analysis of a text, which shows what happens in it. We obtain the paradigmatic analysis of a text by finding the set of bipolar oppositions found (hidden) in the text.

I believe the basic opposition in this joke is between paying for a haircut and not paying for a haircut. We see this opposition in the chart below.

**Table:** 

Pay for Haircut	Don’t Pay for Haircut
The Priest, The Imam	The Rabbi
Ask How Much It Costs	Asks How Much It Costs
Priest and Imam each leave 100 Gold Coins	Rabbi leaves 100 Rabbis

So this joke is about paying for haircuts and the punch line is about the way the Rabbi responded to the barber’s statement that holy men don’t have to pay for haircuts. It reflects an aspect of the Jewish psyche that offers an absurd resolution to the events in the joke. But what is a Jewish humor and what makes a joke a Jewish joke?

## What is Jewish Humor

Freud said he knew of no people who made so much fun of themselves as the Jews and this joke reflects a common Jewish sensibility—to laugh at human foibles, whether they are in lay people or in religious figures like rabbis. This joke is an example of Jewish humor, which we can define as humor in which Jewish people are the main characters and Jewish character traits and culture play an important role in generating the humor.

Avner Ziv, an Israeli humor scholar, defines Jewish humor and explains its origins in Eastern Europe. He writes, in his book *Jewish Humor* (Ziv, 1986, p. 11):

From my standpoint, a Jew is a man who considers himself Jewish and identifies with the Jewish people. Thus, Jewish humor can be defined as humor created by Jews intended mainly for Jews, and which reflects special aspects of Jewish life….Naturally, Jewish humor changes as a result of important changes in the life of the Jewish people. Thus, one can speak of Eastern European Jewish humor, Moroccan Jewish humor, American Jewish humor or Israeli Jewish humor. Nevertheless, what is identified in worldwide professional literature as Jewish humor originated in 19^th^ Century Eastern Europe. There Jews lived under special and extremely harsh conditions confronted with a real danger to their lives. In these conditions, humor developed which had particular characteristics what helped the Jews cope with their terrible ordeals.

The fact that this Jewish joke has an Imam in it, instead of the characters we would find in earlier American jokes about holy men, namely a priest, a protestant minister and a rabbi, reflects important changes that have taken place in American culture and society. America is now a more multi-cultural, multi-ethnic and multi-religious society.

## Conclusions

There are, in this joke, two other holy men: a priest and an imam. But the punch line involves a rabbi and thus I would suggest this is a Jewish joke. Many Jewish jokes involve people from other religions, ethnicities, races, countries, etc. But if the punch line involves Jews, it is generally safe to conclude that we have a Jewish joke. The rabbi in the joke wanted to pay for the haircut, but when the barber told him he didn’t charge holy men for haircuts, the rabbi took advantage of his generosity and sent a hundred other rabbis to the barber. One could argue that the technique of literalness, technique 27, is also at play here since the barber told the rabbi he doesn’t charge holy men for haircuts and that comment led to the punch line in the joke. The punch line, “he found a hundred rabbis,” plays on our expectations that there will be a hundred something in the joke as well as the realization that if the rabbi left a hundred gold coins and nothing else happened there would be no joke. The fact that Jewish people are able to make fun of their rabbis, and often do in their jokes, suggests a different sensibility when it comes to relating to holy men and women (since there are now women rabbis) than you find in many other religions.
